# Ways ahead: protecting, promoting and supporting breastfeeding in the context of HIV

**DOI:** 10.1186/1746-4358-5-19

**Published:** 2010-10-26

**Authors:** Karen Marie I Moland, Penny van Esterik, Daniel W Sellen, Marina M de Paoli, Sebalda C Leshabari, Astrid Blystad

**Affiliations:** 1 Centre for International Health, University of Bergen, Norway; 2 Faculty of Health and Social Sciences, Bergen University College, Norway; 3 Department of Anthropology, York University, Canada; 4 Department of Anthropology, University of Toronto, Canada; 5 Fafo Institute for Applied International Studies, Oslo, Norway; 6 MUHAS, Muhimbili University of Health and Allied Sciences, Tanzania; 7 Department of Public Health and Primary Health Care, University of Bergen, Norway

## Abstract

The HIV epidemic coupled with the assumed benefits of infant formula for the children of all HIV-infected mothers have in complex ways changed public ideas about infant feeding and represents a threat to well established breastfeeding practices. In the wake of the confusion that postnatal prevention of mother to child transmission of HIV (PMTCT) interventions have created among HIV-infected mothers, infant feeding counsellors and the public at large, it is time to reinstate the principles of the Innocenti Declaration to protect, promote and support breastfeeding in the context of HIV. The challenge that lies ahead is a search for ways to restore the trust in breastfeeding as the normal and safest way to feed an infant. This requires continued research as well as concerted advocacy and action.

## Introduction

As a final note, let us return to breastfeeding for a moment and discuss how to counteract the pressures that have been exerted against breastfeeding in the context of HIV. As history shows, the threats to breastfeeding have changed over time. During the last decade the greatest threat to breastfeeding has been the confusion over infant feeding in the wake of the HIV pandemic. Through national and local PMTCT programmes and HIV information campaigns, the global community has learnt that breastfeeding in HIV-infected mothers may be a risk to child survival and should, if possible, be avoided. The uncertainty that this has generated is illustrated in this thematic series. In the early phase of the national and local PMTCT programme implementation, breastfeeding advocacy groups were accused of having their "heads in the sand" about the transmission of HIV through breastfeeding. The existing evidence of the superiority of breastfeeding in terms of infant survival, and the 2010 infant feeding guidelines promoting breastfeeding as the first choice of infant feeding method, have demonstrated that the advocacy groups were right in their firm and concerted action to protect breastfeeding. One lesson is learnt: replacement feeding has substantial negative unintended consequences for the individual mother, for her infant, for households and for health systems. In the aftermath of a decade of trial and error in developing guidelines and implementing postnatal PMTCT programmes, the trust in breastfeeding thus needs to be restored. The challenge is how to 'turn the tide' or change the mindset of PMTCT counsellors, mothers and significant others towards breastfeeding as the safest way to feed an infant. The research studies reported in this thematic series suggest that this may prove challenging given the legacy of efforts to implement earlier guidelines. In the first concluding remarks we focused on global policy documents and lessons learnt [1]. Now in this final paper we consider this challenge in terms of the agreements in the Innocenti Declaration which was adopted in 1991 and reaffirmed in 2005 [2]. The Declaration considered actions around protecting, promoting and supporting breastfeeding that are still valid, and that should be reiterated in the context of HIV.

## Protecting breastfeeding

Protecting breastfeeding remains the most fundamental activity in this regard. According to the Innocenti Declaration women who are already breastfeeding should be protected from influences that might discourage them from continuing to breastfeed such as the promotion of breast milk substitutes [2]. The HIV epidemic coupled with the promotion of infant formula to HIV-infected mothers have changed public ideas about infant feeding and together represent a threat to well established breastfeeding practices. The unethical marketing of infant feeding formula in low income countries in the 1970s was a purely commercial measure, and was counteracted as such by the endorsement of the International Code of Marketing of Breast-Milk Substitutes by the World Health Assembly in WHO in 1981 [3]. In the wake of the HIV pandemic, the promotion of infant formula has been redefined and to a greater extent been justified as a measure to prevent HIV transmission from mother to child. Free infant formula to HIV-infected mothers has in fact been raised as a human rights issue and compared with the fight for free access to antiretroviral treatments (ARVs) [4]. This highly problematic comparison between infant formula and ARVs in human rights discourse has met stark opposition from various sources [5], and feeds into the ongoing debate on choice, on the mother's right to breastfeed and the child's right to be breastfed that the editors of *IBJ*, among others, have engaged in [6,7]. Central to the ethics surrounding the marketing of infant formula is 'the right of the infant to be breastfed' in the sense that no-one may interfere with a mother's right to breastfeed [6,7].

In sub-Saharan Africa however, it is notable that the normative foundation of breastfeeding and the social sanctions expected and experienced by women who are not breastfeeding have been important factors counteracting commercial pressures to formula feed [8-10]. Protecting breastfeeding involves protecting local breastfeeding knowledge and practices from undue interventions. It involves resisting commercial promotion of baby foods in the market and in the health care system by upholding and promoting the Code on the Marketing of Breastmilk Substitutes nationally and locally [3]. In many countries the Code is still not enforced, and adherence remains voluntary. Whenever breastfeeding is threatened new opportunities present themselves for expanding the market for commercial baby foods. Hence '. . . urgent action is required to ensure [that] the principles and aims of the International Code and related resolutions of the World Health Assembly are implemented'[[7] p. 1].

## Promoting exclusive breastfeeding

The promotion of breastfeeding aims to persuade women to breastfeed [[2] p. 5]. In sub-Saharan Africa where breastfeeding remains normative, promotion of breastfeeding is primarily geared towards exclusive breastfeeding, because here as in most parts of the world, exclusive breastfeeding is not customary. Deep-seated cultural factors such as particular traditions attached to the introduction of other fluids and feeds can strongly influence infant feeding practices. But poverty may be an even more important barrier in the promotion of exclusive breastfeeding [7]. It is very difficult for women to succeed in exclusive breastfeeding for six months when they depend on generating an income from work which is added to their domestic chores. They may need to leave the baby with other care-givers from just a few weeks postpartum [11]. Poverty creates situations where there is in fact no choice - one eats what is available, and feeds children what is available, with the support of the partner that is available. Many women also fear or, through bio-behavioural feedback, actually experience breastmilk insufficiency [10-12]. Furthermore, since the implementation of postnatal PMTCT began, exclusive breastfeeding has in many places become associated with HIV-infected mothers. This further complicates its promotion among both HIV-infected and non-infected women [12].

Therefore, a challenge is to promote exclusive breastfeeding as the normal and the safest feeding option for all, as the recent WHO guidelines on HIV and infant feeding now do. The principles of the Baby Friendly Hospital Initiative (launched in 1991 and updated and expanded in 2009) [13], building on the Innocenti Declaration [2], are among the interventions that are planned revitalized in health facilities at all levels in the future.

## Supporting breastfeeding

Exclusive breastfeeding for six months is a great challenge even for mothers who enjoy economic and social security. As Beasley and Amir pointed out in an editorial in this journal, 'As individuals, women are powerless to counter the complexity of social forces that interfere with exclusive breastfeeding their infants for six months' [7]. A major threat to sustain exclusive breastfeeding is the lack of social support. The experiences with postnatal PMTCT programmes so far have clearly demonstrated that in order to succeed in exclusive breastfeeding for six months, support is critical, both economic support to strengthen food security and social support from partners and peers.

Partner involvement in the sense of sharing HIV test results is often seen as a necessary condition for successful adherence to infant feeding recommendations in a PMTCT context. However, disclosure to the partner is often perceived as an ordeal and may not have the intended effect if there is a lack of sensitivity to the women's fear of blame and rejection [8]. Nevertheless, finding new and more effective ways and means to involve partners more systematically and safely needs to be more strongly emphasized in the implementation of the new 2010 guidelines [14].

Programmes for peer support for exclusive breastfeeding critically depend on the establishment of trust, and thus need to take the social and political context into consideration in programme design [15]. Locally adapted breastfeeding support groups modelled after the 'La Leche League' approach could be promoted more widely in sub-Saharan Africa in order to support women's efforts to fight for social and political conditions that facilitate both exclusive and extended breastfeeding for all.

In order to support breastfeeding beyond six months and after complementary feeds are introduced, the concept 'prolonged breastfeeding' emerges as inappropriate since it seems to indicate that breastfeeding takes place for 'too long'. In the editorial of this thematic series and in these final remarks we have replaced 'prolonged breastfeeding' with 'extended breastfeeding' to communicate that this practice is beneficial and should be encouraged and supported.

## Research and advocacy

Protecting, promoting and supporting breastfeeding requires continued research as well as concerted advocacy and action. However, some key questions arise about how research results can be brought forward to new policies, and how researchers and activists, representing different entry points, can join forces and pull together to protect, promote and support breastfeeding.

Drawing implications from research is a challenging task. In the HIV and infant feeding field there are many different actors or disciplines working on the basis of their specific and limited paradigms. While the HIV experts aim to eliminate HIV transmission from mother to child through the elimination of breastfeeding in HIV-infected women, the child survival experts focus on HIV-free survival and advocate for breastfeeding [16]. Miriam Labbok argues for 'transdisciplinarity' in the sense that different disciplines come together "not only to address a predefined problem from each one's perspective, but rather to together define the problem to be addressed" [16]. In order to achieve synergies between different disciplines that influence the HIV and infant feeding policy, she argues that breastfeeding should be seen as a part of the reproductive continuum together with conception, pregnancy, birth and family planning [16]. In practical terms this means an integration of breastfeeding programmes with other reproductive health programmes as exemplified in the revised 'Baby Friendly Hospital Initiative' which, in addition to the 'Ten steps to successful breastfeeding' also include standards and goals for birthing and birth spacing [16].

We support this view on the need for transdisciplinarity in the definition of the problem of HIV and infant feeding. In order to ask the right questions and to reach policy makers on global and local levels with our results, we need to draw upon evidence from different disciplines and use different kinds of methodological approaches including epidemiology, biomedicine and ethnography. We need hard facts and numbers, but we also need narratives documenting the experiences of particular people including mothers and fathers, peer counsellors and health workers.

However neither stories nor numbers generate policy change by themselves. In order to make an impact on infant feeding policy, researchers need allies within breastfeeding advocacy groups that can effectively utilise research results in their advocacy work. Actors like WABA (World Alliance for Breastfeeding Action) which takes its mission from the Innocenti Declaration; IBFAN (the International Baby Food Action Network) that takes its mandate from the Code on the Marketing of Breastmilk Substitutes; and La Leche League and other mother support groups that provide practical help and support for new mothers who want to breastfeed, have long traditions with research-activist collaboration [17]. There may further be a need for the research community to create allies across social movements; with the women's health movement, and with sexual and reproductive rights-, environmental-, and human rights movements [16-18].

But when working across these single issue causes, we need to be aware of potential conflicts of interests. Advocacy groups can move fast, but because they are ideologically driven they can make counterfactual errors. Recent examples that involve unanticipated threats to breastfeeding by advocacy groups include the labelling of breast milk "the world's most polluted food" by Greenpeace [19], the characterization of breastfeeding as a "cause" of HIV infections by HIV education campaigns, and the demand for free infant formula as a human right by treatment advocacy groups. In order to prevent such counterfactual errors, the research community should to a greater extent take on the responsibility to ensure that advocacy groups are working on the basis of up-to-date and unbiased knowledge.

## A final remark

During the symposium in Rosendal, participants noted that the postnatal PMTCT initiative has brought western and public health biases to local practices that have been adaptive. Lactation is an ancient adaptation in mammals that has been shaped by evolution [20]. Indeed, recent analyses of the continued relevance of the evolutionary forces shaping breastfeeding highlight the fact that it has never been lost to any species and that the same functional benefits of breastfeeding for infants and mothers persist in humans as in all other mammals [20]. The scientific evidence base warns against hasty dismissal of the evolved benefits of breastfeeding. The experiences of postnatal PMTCT interventions add substance to this stand. In future, the global health professional community should be more sceptical of claims about the risks of breastfeeding.

The challenge that lies ahead is to search for ways that will protect, promote and support breastfeeding in ways that will reinstate the trust in breastfeeding as the normal and safest way to feed all infants. The 2010 WHO HIV and infant feeding guidelines support this trajectory.

## Competing interests

The authors declare that they have no competing interests.

## Authors' contributions

KMIM and AB wrote the first draft of the paper drawing heavily on PVE's summing-up paper from the workshop. DWS and MMDP contributed to subsequent drafts. SCL provided important inputs in the planning phase. All authors approved the final paper.
